# Impact of physical exercise participation on socioeconomic status: an empirical study using CGSS data

**DOI:** 10.3389/fpubh.2025.1645125

**Published:** 2025-10-06

**Authors:** Chenlei Zhao, Junhua Zhou, Mengmeng Man

**Affiliations:** School of Physical Education, Ludong University, Yantai, China

**Keywords:** physical exercise, SES, health status, individual income, mediating effect

## Abstract

**Introduction:**

Recently, China’s rapid economic development and significant improvements in residents’ quality of life have led to the integration of physical exercise into the daily routines of the general public. While promoting health, this trend may have a far-reaching impact on individuals’ socioeconomic status (SES) through the accumulation of human, social, and economic capital. Can participation in physical exercise improve SES? If so, what are the mechanisms of influence? Are there differences among various groups?

**Method:**

To address these questions, this study utilized data from the 2021 China General Social Survey to construct a regression model for investigation.

**Results:**

This study found that individuals with higher SES demonstrate greater awareness of and participation in physical exercise. Participation in physical exercise significantly increases the probability of individuals improving their SES, but the effect varies by gender, urban or rural residence, marital status, education level, and generation. Specifically, males, urban residents, married individuals, those with lower education levels, and older individuals benefit more. Mechanistic analysis suggests that health status and personal income are key mediators, with physical exercise enhancing SES through improved health and higher income.

**Discussion:**

This study can help individuals understand how physical exercise can improve their SES. It serves as a reference for policymakers aiming to promote the coordinated and sustainable development of physical exercise and socioeconomics across different geographic regions and urban and rural environments, supporting individuals in their pursuit of higher SES.

## 1 Introduction

Recently, China’s economy has experienced significant growth, with remarkable improvements in social productivity, comprehensive national strength, and residents’ living standards. In 2021, the State Council issued the National Fitness Program (2021–2025), accelerating the integration of physical exercise and fitness awareness into daily life. As a result, an increasing number of individuals now view regular physical exercise as an indispensable component of their lifestyles. As a fundamental aspect of human society, sports embody a dialectical unity of intrinsic attributes and derived values (1). Sports inherently enhance physical health and emotional well-being through bodily movement. However, their functions have expanded within social development, generating multidimensional values, including economic growth and social integration (2). Nobel laureate Robert W. Fogel’s seminal study revealed that approximately half of the economic growth during the Nordic Industrial Revolution originated from productivity gains driven by improvements in population health. This finding illuminates the historical logic underlying the transformation of sports’ intrinsic functions into economic benefits. In contemporary society, individual participation in physical exercise not only facilitates healthy lifestyles but also significantly impacts socioeconomic status (SES) through the accumulation of human, social, and economic capital.

SES, a pivotal concept in social stratification research, refers to an individual’s composite position within societal hierarchies determined by disparities in social resources (3), such as income level, educational attainment, and occupational prestige (4). As a multidimensional metric integrating social, educational, and material capital, SES reflects an individual’s resource allocation within the stratification system (5). The health, social, and capital-related benefits derived from physical exercise are essential prerequisites for enhancing SES. However, existing research has lacked a systematic empirical examination of the specific mechanisms through which physical exercise influences SES and the heterogeneous effects across demographic groups. Amidst deepening national fitness strategies and evolving social stratification structures, scientifically evaluating the association between physical exercise and SES requires both theoretical reasoning and large-scale, data-driven quantification.

In this context, this study used data from the 2021 China Comprehensive Social Survey to construct a regression model exploring the relationship between physical exercise participation and individual SES. Through rigorous data processing and model analysis, this study examined how physical exercise influences individual SES, providing a new perspective on the interaction between physical exercise and SES. This study also analyzed the heterogeneity of the impact of physical exercise on individual SES, enriching the theoretical framework on the relationship between physical exercise and SES by comparing differences across gender, urban and rural areas, marital status, education level, and generational status. This finding not only helps individuals better understand the logic and motivation behind using physical exercise to improve their SES, enabling them to formulate more scientific and reasonable exercise plans but also provides valuable insights for social policymakers. These insights can promote the coordinated and sustainable development of sports and social economy across regions and environments, better meeting individuals’ aspirations for higher SES.

This study is divided into six parts. The first part introduces the study’s background. The second reviews the literature on the impact of physical exercise on physical health and individual income, which in turn affect SES, and presents three hypotheses. The third part describes the study design, including data sources, variable selection, and research methods. The fourth part analyzes the empirical results. The fifth part presents the conclusions and policy recommendations. The sixth part discusses the study’s limitations and prospects.

## 2 Literature review and research hypotheses

### 2.1 Direct effect of participation in physical exercise and individual SES

Physical exercise involves using physical activities and combining natural forces to improve health and strengthen physical fitness. This study specifically focuses on non-competitive, non-school physical exercise undertaken by residents for health purposes. SES encompasses family, community, and individual levels, but this study focused on the impact of physical exercise on individual SES.

As a form of positive health investment, physical exercise can directly improve individual SES through multiple pathways. From a behavioral economic perspective, physical exercise is fundamentally a process of optimizing human capital. Regular, long-term participation in physical exercise not only promotes a healthy physique and positive mental outlook but also creates an external image advantage that exerts a significant “primacy effect” on career development (6). Moreover, personality traits, such as self-discipline and perseverance, developed through physical exercise, are crucial for career advancement. Studies have indicated that employees who regularly participate in physical activities demonstrate stronger resilience during the project execution stage. This enhanced competitiveness from physical exercise can directly support individuals in advancing their careers, thereby significantly improving their SES (7). Seippel, O. explored the relationship between sport and social status among young individuals in Norway, finding that sport generally holds a high status, especially among sports participants, and that it confers higher social status on boys (8). Zhou, C. found that residents’ participation in physical exercise has a significant positive impact on their class identity (9), a finding also confirmed by Hu, R. (10), who highlighted that physical exercise is a class-distinctive behavior (11). Xing, X. explored the correlation between physical participation and SES, discovering that individuals with higher income, educational, and professional status are more inclined to participate in physical exercise. High SES groups also have high access to sports resources, as well as more time and money for participation. This difference is reflected not only in the participation rate but also in the frequency and type of physical activities (12). In addition, family economic status can directly affect adolescents’ physical exercise behavior and indirectly affect them through parental behaviors (13, 14). Eakins, J. found that household SES played a decisive role in determining physical exercise participation and sports expenditure during the recession in Ireland (15). Similarly, Richard, V. revealed that individuals with higher SES are more likely to participate in physical exercise more frequently (16). All these studies suggest that SES can influence an individual’s participation in physical exercise. Conversely, can it be assumed that an individual’s participation in physical exercise leads to an increase in their SES? Based on the above discussion, Hypothesis 1 is proposed.

*H1*: Participation in physical exercise significantly increases the probability of an individual’s SES.

### 2.2 Mediating role of physical health status and individual income on individual SES

New data from the World Health Organization indicates that approximately 31% of adults worldwide did not meet the recommended level of physical exercise in 2022. If this trend continues, it is expected to rise to 35% by 2030 (17). Lack of physical exercise can increase the risk of cardiovascular disease and diabetes in adults. In contrast, regular physical exercise can enhance cardiopulmonary function, improve immunity, and reduce the risk of chronic diseases, including cardiovascular disease and diabetes (18). The mental health and well-being of individuals who regularly participate in physical exercise are better than those who do not, especially among college students and the older adults (19, 20). Guo, M. also revealed that SES has a lasting impact on individual health and does not change with age (21). Wang, F. empirically studied the relationship between physical exercise and individual health, finding that physical exercise can significantly improve individual health, especially for the subjective and objective health status of middle-aged and older adults (22). In another study, she analyzed the national health and welfare development trend in China over the past 20 years, examining the coordination between the health and welfare systems. She also calculated the contribution of physical exercise to national health and welfare, reporting a total contribution rate of 3.79% (23). Sweeting, H. studied the association between adolescent social status and several health measures, revealing that school status was more strongly correlated with health than SES. Moreover, subjective SES was associated with health even after adjusting for objective SES (24).

There is a strong correlation between participation in physical exercise and household income and expenditure (25). Higher income positively impacts both time and money allocated to physical exercise (26), with income and sports expenditure increasing in tandem (27). The type of physical exercise and the motivation behind it are key determinants of spending (28). In family life, the higher the SES of parents, the more they invest in their children’s participation in sports clubs (29). Additionally, income and education levels are almost positively correlated with sports expenditure (30).

However, does participating in physical exercise increase an individual’s economic income? Based on data from the China Labor Force Dynamics Survey and the China General Social Survey, Zhong, H. empirically examined the impact of participating in physical exercise on residents’ income. The results indicated that participating in physical exercise has a significant positive impact on residents’ income (31). Wang, X. explored the impact of physical exercise on employee performance in their study, with results revealing that participating in weekend physical exercise positively influences employees’ work performance in the following week by improving their positive experiences (32). Consequently, it can be considered that physical exercise not only improves physical health but also increases individual income. Based on this discussion, the second hypothesis of this study is proposed.

*H2*: Participation in physical exercise has a significant positive effect on individual health and income.

Healthy individuals can take on higher-intensity work tasks and have an advantage in professional competition, resulting in higher incomes and better career development opportunities. Health capital influences labor supply, income, and consumption for residents and their families, significantly changing the household balance and life satisfaction, which in turn affects residents’ subjective class identity (33, 34). Therefore, can participation in physical exercise improve one’s SES by improving one’s health? The increase in individual income further strengthens the role of physical exercise in improving SES. Higher income grants individuals access to more social resources, such as high-quality education, medical services, and high-end social activities. The accumulation of these resources helps individuals ascend in the social stratification system. Additionally, the increase in income provides economic security, enabling individuals to continue investing in physical exercise. Zhuang Jiachi used data from the CGSS2017 to explore the wage premium effect of physical exercise. After controlling for individual SES and other characteristics, the study found that regular physical exercise was associated with a 7.36% wage premium, meaning that regular participation in physical exercise leads to a positive increase in wage income (35). Some studies have revealed that both absolute and relative income significantly positively impact residents’ happiness (36), which in turn has a positive impact on SES (37). The higher the individual income, the greater the possibility of self-rated SES (38). Consequently, can participation in physical exercise improve one’s SES by increasing income? Based on the above discussion, the third hypothesis of this study is proposed.

*H3*: Participation in physical exercise enhances individuals’ SES by improving their health and income.

**Figure 1:**
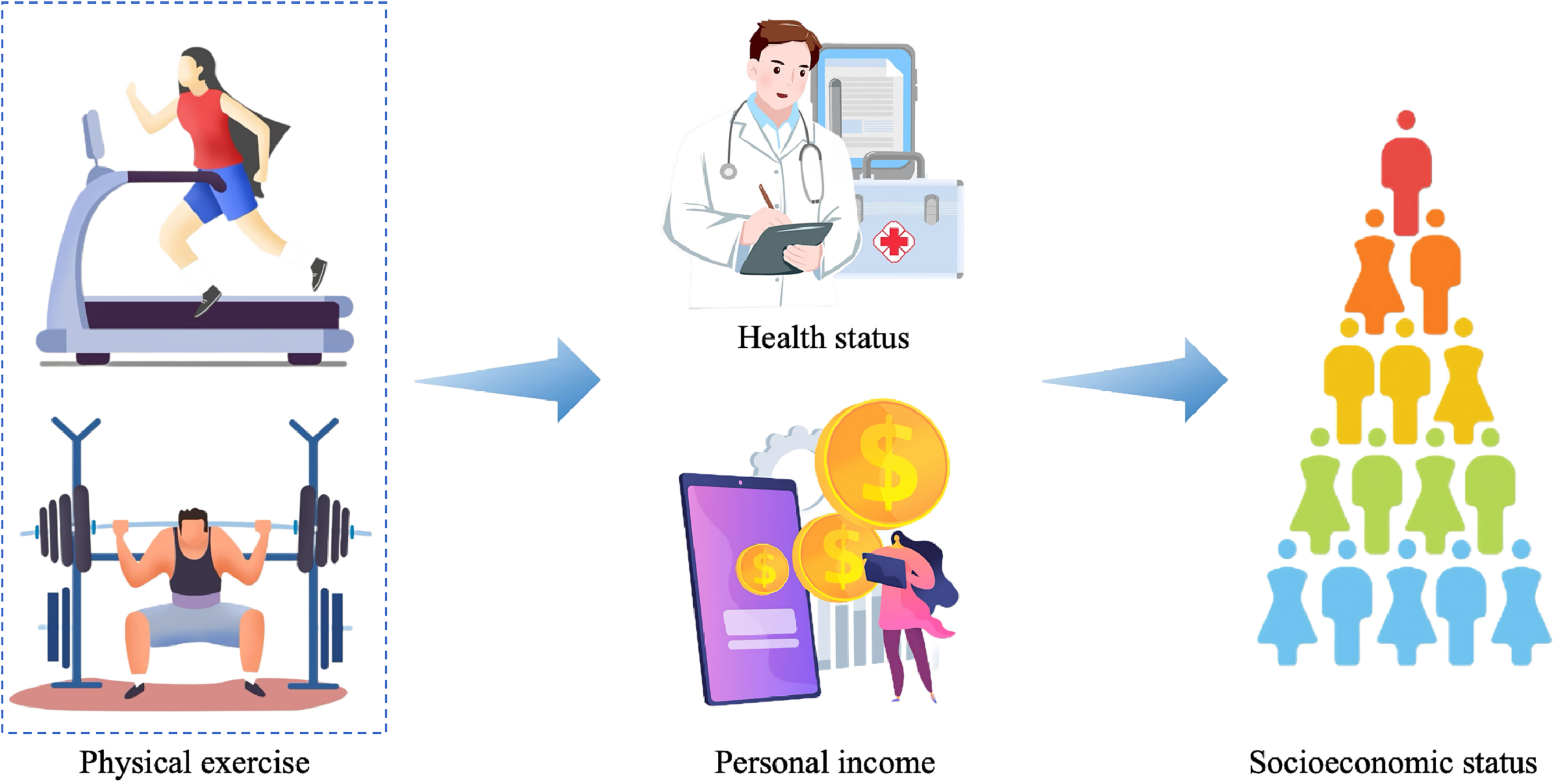
The mechanism by which participating in physical exercise enhances SES.

Figure 1 illustrates Hypothesis 3, helping readers visualize how physical exercise exerts an impact on SES through a mediating effect.

## 3 Research design

### 3.1 Data source

CGSS is an open-source database led by the Chinese University of China. In 2021, it conducted a sample survey across 21 provinces (including autonomous regions and municipalities directly under the central government) in mainland China, excluding Hong Kong, Macao, and Taiwan. The data from the Xinjiang Uygur Autonomous Region, Tibet Autonomous Region, Yunnan Province, Guangdong Province, Guizhou Province, Hainan Province, Heilongjiang Province, Jilin Province, Tianjin Municipality, and Shanghai City were not included in the statistics. The questionnaire is well-structured, and the survey sample is detailed, reflecting the real socioeconomic conditions of Chinese residents. The questions in the questionnaire were quantified using a Likert scale. The China Survey and Data Center tested the reliability and validity of the samples, resulting in 8148 samples. CGSS2021 data were then screened using Stata 17.0 software, and after excluding the samples with incomplete questionnaire answers and missing values according to the selected independent, dependent, control, and mediating variables, 5,410 valid samples were obtained. Missing values mainly fell into three categories: “Other” “Do not know” and “Refused to answer.” Given that missing values may affect the final results, any individual with responses belonging to any of these three categories across all variables was excluded from the statistical analysis, with their full dataset removed.

### 3.2 Variable selection

The dependent variable chosen in this study was SES. SES, also known as social class, refers to the material resources individuals possess within the social class structure and their perception of their own social and economic status, which can be categorized as either subjective or objective (39). Following the research method of Yuan, Q. (40), this study mainly focuses on subjective SES. Subjective SES is an individual’s perception of their SES compared to others, representing a self-assessed evaluation (41). In this study, subjective SES is more consistent with individuals’ actual perceptions of their social position. Selecting this indicator offers a more direct reflection of individuals’ real feelings in horizontal social relationships (when compared to peers), and these feelings themselves constitute a crucial dimension of SES. Additionally, using subjective SES as the dependent variable avoids the problem of inconsistent measurement standards that is associated with objective SES indicators. This variable is an ordinal multi-categorical variable. The corresponding questionnaire item is A43e, which asks “On the whole, what is your SES in the current society?” The independent variable selected for this study is participation in physical exercise, with the corresponding questionnaire item being A30_9, which asks “In the past year, have you regularly participated in physical exercise in your free time?” To control for the influence of other factors on the dependent variable, this study selected the following control variables, divided into three parts. The first set includes demographic characteristics, including gender, age, marriage, political outlook, education level, social class, and basic medical insurance. The second set is the regional variable, which includes household registration (classified as urban or rural) and region (classified as western, central, and eastern regions according to the National Bureau of Statistics). The third set includes other control variables related to this study, including household economic level and number of houses. Based on the study’s proposed hypothesis, health status and individual income were selected as mediating variables. The questionnaire item for assessing an individual’s health status is A15, which asks “How would you describe your current physical condition?” The item for personal income is A8a, asking “What was your total personal income last year?“The names, source items, definitions, and descriptive statistics of all variables are presented in Table 1.

**Table 1:** Variable names, definitions, and descriptive statistics.

Variable name and source item	Variable definitions	Maximum	Minimum	Average value	Standard deviation
Dependent variable					
SES A43e	Lower layer = 1, middle lower layer = 2, middle layer = 3, upper middle layer = 4, upper layer = 5	1	0	2.316	0.891
Independent variable					
Physical exercise A30_9	Attend = 1, Absent = 2	1	0	0.674	0.469
Control variable					
GendersA2	Male = 1, Female = 0	1	0	0.507	0.500
Household registration A18	Urban household = 1, Agricultural household = 0	1	0	0.456	0.498
Age A3	Year	95	18	52.513	16.440
Marriage A69	Married = 1, Unmarried = 0	1	0	0.742	0.438
Member of the Chinese Communist Party A10	party members = 1, non-party members = 0	1	0	0.146	0.353
Educational attainmentA7a	Primary and below = 1, Junior high school = 2, Senior high school (Technical secondary school, Technical school) = 3, University specialist = 4, Higher Junior College = 5	5	1	2.459	1.336
Household economic status A64	Well below average = 1, below average = 2, average = 3, above average = 4, well above average = 5	5	1	2.618	0.768
Number of properties A65	Quantity	11	0	1.238	0.699
Social hierarchy A43_a	Lower class = 1, Lower middle class = 2, Middle class = 3, Upper middle class = 4, Upper class = 5	5	1	2.486	0.926
Basic medical insurance A61_1	Enroll = 1, Non-enroll = 2	1	0	0.952	0.0213
Region S41	West = 1, Central = 2, East = 3	3	1	2.168	0.822
Mediation variables					
Health status A15	Very unhealthy = 1, relatively unhealthy = 2, fair = 3, relatively healthy = 4, very healthy = 5	5	1	3.511	1.054
Personal income A8a	Ln (Gross personal income)	16.117	2.996	10.120	1.364

Data source: Compiled from the 2021 Chinese General Social Survey data.

### 3.3 Research method

The dependent variable in the study is “subjective SES,” an ordered categorical variable ranging from 1 to 5. The Ologit model is specifically designed for ordered categorical dependent variables, enabling it to capture the hierarchical relationships among categories, rendering it more suitable for the present data characteristics than ordinary multiple linear regression or unordered categorical Logit models. In this study, we adopted Hu, D’s research method and used the Ologit model to analyze the impact of physical exercise on SES (42), establishing a benchmark model (Equation 1) as follows:


(1) $${\mathrm{SES}}_{i}=\alpha_{0}+\alpha_{1}{\text{excercise}}_{i}+\alpha_{x}{\text{control}}_{i}+\varepsilon_{i}$$

Model (1) includes *SES_i_* as subjective SES, *exercise_i_* as participation in physical exercise, and *control_i_* as relevant control variables. *α*_1_ is the parameter to be estimated.

The mediating effect mechanism suggests that participation in physical exercise does not directly affect SES but indirectly influences it through relevant mediating variables. Therefore, to further validate the mediating roles of health status and personal income in the promotion of SES, a step-by-step test method was used to establish the models (Equations 2,3) (43).


(2) $${\text{mediation}}_{i}=\beta_{0}+\beta_{1}{\text{excersise}}_{i}+\beta_{x}{\text{control}}_{i}+\varepsilon_{i}$$

(3) $${\mathrm{SES}}_{i}=\delta_{0}+\delta_{1}{\text{excercise}}_{i}+\delta_{2}{\text{mediation}}_{i}+\delta_{x}{\text{control}}_{i}+\varepsilon_{i}$$

Mediation_i_ is a mediating variable, including health status and personal income. In model (1), α1 is the total effect of physical exercise on SES after controlling for basic variables. In model (2), β1 is the coefficient indicating the effect of physical exercise on the mediating variables after controlling for the basic variable. In model (3), δ1 and δ2 are coefficients representing the effects of physical exercise and the mediating variables on SES after controlling for basic variables. For the mediation mechanism to be established, α1, β1, and δ2 must be significant. In this case, if δ1 is not significant, it indicates a complete mediating effect. If δ1 is significant and δ1 < α1, it indicates a partial mediating effect.

## 4 Analysis of empirical results

### 4.1 The state of sports exercise among individuals with different socioeconomic statuses

**Figure 2:**
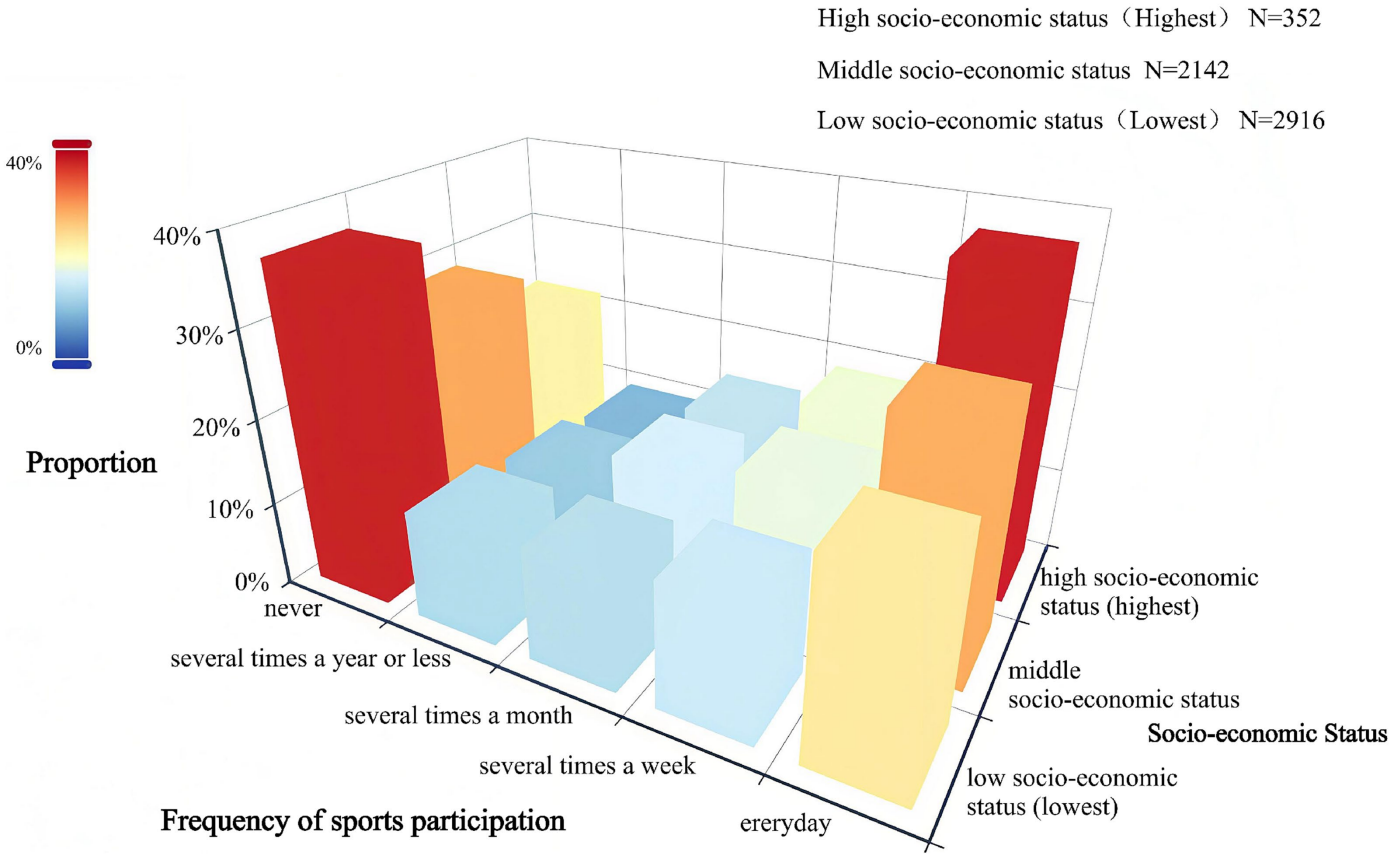
Frequency distribution of participation in sports exercise by individuals of different SES.

Table 2 indicates that the proportions of individuals with low, medium, and high SES who never participated in physical exercise were 36.97, 28.38, and 21.59%, respectively, indicating an overall downward trend. In contrast, the proportions of individuals with low, medium, and high SES who participated in physical exercise were 22.50, 27.87, and 36.93%, respectively, indicating an overall upward trend. Figure 2 clearly illustrates that the proportion of individuals with lower SES who never participate in physical exercise and the proportion of individuals with higher SES who participate in physical exercise every day are at the two highest points. Additionally, as SES increases, the probability of individuals participating in physical exercise rises. The chi-square value was 90.908 (*p* < 0.001), indicating significant differences in the frequency of physical exercise among individuals with different SES levels. This analysis exhibits that higher SES is associated with greater awareness of physical exercise and more active participation.

**Table 2:** Distribution of physical exercise frequency across different SES groups.

Status	Low SES (Lowest)	Middle SES	High SES (Highest)
Frequency			
Never	36.97%	28.38%	21.59%
Several times a year or less	12.76%	11.06%	9.38%
Several times a month	13.13%	15.36%	13.92%
Several times a week	14.64%	17.32%	18.18%
Everyday	22.50%	27.87%	36.93%

### 4.2 Examination of the effect of participation in physical exercise on the promotion of SES

To test the effect of physical exercise on improving individual SES, Ologit regression was performed with SES as the dependent variable, incorporating independent, control, and mediating variables according to the established model (1).

In Table 3, Model (1) included only physical exercise as the explanatory variable, and the results indicated a coefficient of 0.606 (*p* < 0.01), indicating that participation in physical exercise significantly improves individual SES without controlling for other factors. The odds ratio value was 1.833, meaning that the probability of individuals participating in physical exercise improving their SES by one level was 83.3% higher than those who did not participate, providing preliminary support for the basic model. After introducing independent and control variables in the model (2), the regression coefficient decreased to 0.193 but remained significant (*p* < 0.01). This suggests that the effect of physical exercise on SES remains robust even after accounting for multiple socio-demographic characteristics. The odds ratio was 1.213, indicating that, after controlling for other variables, individuals who participated in physical exercise were 21.3% more likely to achieve a one-level increase in SES than those who did not participate. Models (1) and (2) collectively demonstrate that participation in physical exercise significantly increases the probability of individuals improving their SES, thus validating hypothesis 1.

**Table 3:** Ologit regression results for SES improvement through physical exercise.

Variable	SES			
	(1)	(2)	(3)	(4)
Physical exercise	0.606^***^	0.193^***^	0.182^***^	0.158^**^
	(11.198)	(3.019)	(2.843)	(2.442)
Genders		−0.104^*^	−0.115^**^	−0.176^***^
		(−1.881)	(−2.080)	(−3.131)
Household registration		0.170^***^	0.174^***^	0.068
		(2.666)	(2.733)	(1.024)
Age		0.005^**^	0.007^***^	0.011^***^
		(2.275)	(3.155)	(4.609)
Marriage		0.134^**^	0.130^**^	0.093
		(2.122)	(2.052)	(1.467)
Communist Party		0.018	0.012	−0.015
		(0.210)	(0.140)	(−0.176)
Educational attainment		−0.006	−0.007	−0.033
		(−0.216)	(−0.247)	(−1.111)
Household economic status		1.021^***^	1.002^***^	0.969^***^
		(23.489)	(22.868)	(21.917)
Number of properties		0.089^**^	0.092^**^	0.086^**^
		(2.220)	(2.305)	(2.147)
Social hierarchy		0.715^***^	0.709^***^	0.705^***^
		(36.939)	(36.469)	(36.218)
Basic medical insurance		0.279^**^	0.289^**^	0.275^**^
		(2.108)	(2.187)	(2.077)
Region		0.087^**^	0.082^**^	0.037
		(2.491)	(2.321)	(1.022)
Health status			0.105^***^	0.089^***^
			(3.583)	(3.009)
Personal income				0.162^***^
				(5.968)
Prob>Chi^2^	126.298	3265.198	3278.058	3314.787
Pseudo R^2^	0.009	0.240	0.241	0.244
Observation	5,410	5,410	5,410	5,410

z-statistics in parentheses.

*p < 0.1, **p < 0.05, ***p < 0.01.

In model (3), the health status variable was added to model (2), causing the regression coefficient for physical exercise to decrease slightly to 0.182 while its significance remained unchanged (*p* < 0.01). The regression coefficient for health status was 0.105 (p < 0.01), indicating that improved health status also contributed to SES improvement, suggesting potential mediating effects. In model (4), individual income variables were added to capture the explanatory power of the economic dimension. The regression coefficient for physical exercise dropped to 0.158, and its significance was slightly weakened (*p* < 0.05) but statistically significant. The results revealed that the positive effect of physical exercise persisted even after controlling for income level, and personal income had a significant positive impact on SES (0.162, *p* < 0.01), consistent with expectations. Models (3) and (4) suggest that the effect of physical exercise on individual SES may occur through improvement in individual health and increases in personal income. Regarding goodness-of-fit, the Pseudo R^2^ of the model gradually increased from 0.009 to 0.244, indicating that the model’s explanatory power increased with the inclusion of control variables. Notably, the overall explanatory power of the model significantly increased after adding variables, such as household economic level and social class.

### 4.3 Heterogeneity analysis of participation in physical exercise to enhance SES

To further explore whether the impact of physical exercise on SES differs among population subgroups, the results of grouped ordered Ologit regression by sex, urban and rural household registration, and marital status are presented in Table 4, with all models controlling for the variables listed above. The analysis of gender heterogeneity indicates that participating in physical exercise significantly improved male SES (0.291, *p* < 0.01) but did not significantly affect female SES (0.106, *p* > 0.1). The results indicate that the socioeconomic returns of participating in physical exercise are more pronounced among men, potentially due to the social recognition, career opportunities, or network resources they acquire through such engagement. The rise of women’s status in China has closely followed a series of social revolutions and reforms in modern China. Particularly over the past four decades since the reform and opening-up, women have assumed increasingly significant roles in Chinese society. While gender equality has advanced noticeably in areas like education and culture, progress in sports has been far less satisfactory. Women’s sports rights have long been inadequately prioritized, leading to a lack of recognition for their athletic entitlements and inadequate societal attention (44). This is primarily reflected in unequal distribution of sports resources (45), constraints imposed by traditional notions, and limited choices in sports disciplines (46). As a result, women face greater barriers than men when seeking to improve their SES through participation in physical exercise.

**Table 4:** Gender, urban-rural, and marital differences in SES promotion through physical exercise participation.

Variable	SES					
	Male	Female	Town dwellers	Rural dwellers	Married	Unmarried
Physical exercise	0.291^***^	0.106	0.329^***^	0.130	0.237^***^	0.034
	(3.196)	(1.169)	(3.017)	(1.642)	(3.257)	(0.247)
Control variables	Control	Control	Control	Control	Control	Control
Prob > Chi^2^	1793.309	1483.955	1612.188	1516.818	2382.186	884.689
Pseudo R^2^	0.258	0.223	0.269	0.205	0.237	0.250
Observation	2,743	2,667	2,468	2,942	4,012	1,398

z-statistics in parentheses.

^***^p < 0.01.

The urban–rural regression result reveals that participation in physical exercise significantly improves the SES of urban residents (0.329, *p* < 0.01) but does not have a significant effect (0.130, *p* > 0.1) among rural residents. This phenomenon reflects the inequality in sports resources between urban and rural areas. Urban areas are equipped with more comprehensive public sports facilities and diverse sports services, and urban residents’ sports activities often integrate into broader social networks, such as gyms, badminton clubs, and marathons. In contrast, rural areas face relatively scarce sports resources, with inadequate and scattered facilities. Physical exercise in rural areas typically occurs within familiar social circles (neighbors, relatives), featuring a higher degree of homogeneity among participants. Consequently, compared with urban residents, rural residents find it more difficult to improve their SES through physical exercise.

This may be attributed to the fact that marital status revealed that participation in physical exercise had a significant effect on the SES of the “married” group (0.237, *p* < 0.01) but no significant effect on the “unmarried” group (0.034, *p* > 0.1). This may be because marital relationships provide a more stable social support network, facilitating the externalization of the effects of physical exercise into social recognition or economic gains. In contrast, the social networks of “unmarried” group tend to be relatively loose, and the consistency of their exercise routines is easily disrupted by factors such as job changes and lifestyle rhythms. Consequently, the impact of physical exercise on their socioeconomic status is limited.

To explore the differences in the social returns of physical exercise among groups with different education levels, the results of ordinal Ologit regression grouped by education level are presented in Table 5. Each model controls for demographic variables, family background, and regional characteristics, with subjective SES as the explanatory variable. The results indicated that physical exercise significantly improves the SES of individuals with a primary education level or below (0.288, *p* < 0.01), indicating that physical exercise may serve as an important alternative pathway for improving social identity and economic performance among those with low educational attainment. The results are consistent with the notion that individuals with lower education are more dependent on manual labor or face higher barriers to accessing social resources, making physical exercise a more marginal means of improving personal image and social capital. The regression coefficients of physical exercise for the other education groups were 0.124, 0.085, and 0.487, respectively, none of which were statistically significant (*p* > 0.1). However, in the group with a bachelor’s degree or above, the regression coefficient of physical exercise on SES was negative (−0.348) and not significant (*p* > 0.1). The results reveal that the determination of social status among highly educated individuals depends more on professional ability, educational capital, economic capital, or accumulation of social resources, rendering the marginal effect of physical exercise relatively limited. In contrast, individuals with lower educational attainment, who lack sufficient educational capital, often have to compensate for their deficiencies in human capital, social capital, and economic capital through physical exercise. Overall, the impact of physical exercise on SES was more significant in the low-education group, exhibiting a marginal decreasing trend (47). Overall, the impact of physical exercise on SES was more significant in the low-education group, displaying a marginal decreasing trend.

**Table 5:** Educational attainment differences in SES promotion through physical exercise.

Variable	SES				
	Primary and below	Junior high school	Senior high school	Higher Junior College	Undergraduate and above
Physical exercise	0.288^***^	0.124	0.085	0.487	−0.348
	(2.787)	(1.183)	(0.541)	(1.547)	(−0.869)
Control variables	Control	Control	Control	Control	Control
Prob > Chi^2^	877.340	870.020	728.108	269.124	439.536
Pseudo R^2^	0.218	0.219	0.284	0.249	0.261
Observation	1,589	1,607	1,060	448	706

z-statistics in parentheses.

^***^p < 0.01.

To further investigate the differential role of physical exercise in improving SES across different birth generations, the samples in Table 6 were divided into five groups according to birth age: pre-60s, post-60s, post-70s, post-80s, and post-90s. Ordered Ologit regression estimation was then performed, with all models controlling for individual demographic characteristics, family background, health status, and regional factors.

**Table 6:** Generational differences in SES promotion through physical exercise.

Variable	SES				
	pre-60s	post-60s	post-70s	post-80s	post-90s
Physical exercise	0.297^***^	0.253^*^	0.032	−0.052	−0.020
	(2.907)	(1.923)	(0.206)	(−0.285)	(−0.078)
Control variables	Control	Control	Control	Control	Control
Prob>Chi^2^	1168.454	762.638	529.185	459.571	340.215
Pseudo R^2^	0.248	0.261	0.233	0.235	0.210
Observation	1799	1,144	928	842	697

z-statistics in parentheses.

^*^p < 0.1, ^***^p < 0.01.

The empirical results indicate that physical exercise has a significant positive effect on the SES of the early birth group (pre-60s and post-60s). Specifically, the coefficient of physical exercise participation in the pre-60 group was 0.297, which was significant at the 1% confidence interval, indicating that participation in physical exercise significantly improved SES for this generation. In the post-60s group, the effect remained significant (coefficient 0.253, *p* < 0.1). Although the significance decreased slightly, this can be attributed to the fact that, in a traditional social context, participation in physical exercise is more likely viewed as a symbol of individual motivation, self-discipline, or social adaptability, which in turn enhances social evaluation and economic returns. However, from the post-70s generation onward, the positive effect has weakened significantly or even become insignificant. In the post-70s group, the regression coefficient of physical exercise was 0.032 (*p* > 0.1), whereas in the post-80s and post-90s groups, the coefficients were −0.052 and −0.020, respectively, neither of which reached statistical significance. This pattern indicates that, over time, the marginal effect of physical exercise on SES has revealed a decreasing trend.

Most individuals born before 1960 and those born between the 1960s and early 1970s have already retired, affording them more time for physical exercise. This enables them to address deficiencies in their health and social capital, thereby enhancing their SES. Those born in the 1970s and 1980s grew up during a period of rapid societal transformation. Specifically, many are employed in declining traditional industries and struggle to meet the knowledge and skill demands of emerging sectors, frequently facing career development bottlenecks. Additionally, they are caught between caring for parents and raising young children, bearing heavy family responsibilities and high expenses. Even if they engage in physical exercise, it remains difficult to alleviate the economic pressures and declining social status arising from these professional and family challenges. Meanwhile, individuals born in the 1990s grew up amid widespread internet access and the rise of social media. Their accumulation of social capital relies more heavily on social and professional networks and educational capital. Their social interactions are increasingly fragmented across digital platforms, diluting the social function of physical exercise. Consequently, they find it hard to directly improve their SES through sports participation. The aforementioned differences in educational attainment further corroborate this perspective.

The above empirical results reveal that the effects of physical exercise on improving individual SES vary by gender, urban–rural status, marital status, education level, and generation. Male individuals, urban residents, married individuals, those with a low educational background, and older individuals are more likely to benefit from participating in physical exercise to improve their SES.

### 4.4 Test of the mechanism of participation in physical exercise to enhance SES

To further identify the mechanism through which participation in physical exercise affects individual SES, this study constructed a mediation analysis model to examine two potential pathways: health status and individual income. The combined regression results from the ordinal Ologit and linear regression models are presented in Table 7. In model (1), the total effect of physical exercise on SES was significantly positive (coefficient 0.193, *p* < 0.01), establishing a basis for the mediation effect test. Models (2) and (3) constitute the mediation model group of the “health status pathway,” and models (4) and (5) constitute the mediation model group for the “individual income pathway.”

**Table 7:** Testing the mediating effect of health status and personal income.

Variable	SES	Health status	SES	Personal income	SES
	(1)	(2)	(3)	(4)	(5)
Physical exercise	0.193^***^	0.156^***^	0.182^***^	0.223^***^	0.173^***^
	(3.019)	(2.649)	(2.843)	(4.067)	(2.696)
Health status			0.105^***^		
			(3.583)		
Personal income					0.169^***^
					(6.269)
Control variables	Control	Control	Control	Control	Control
Prob > Chi^2^	3265.198	1089.010	3278.058	3544.673	3304.767
Pseudo R^2^	0.240	0.071	0.241	0.081	0.243
Observation	5,410	5,410	5,410	5,410	5,410

z-statistics in parentheses.

^***^p < 0.01.

The test results of models (2) and (4) revealed that the regression coefficients (β1) of physical exercise on health status and individual income were 0.156 and 0.223, respectively, both significant at the 1% confidence interval. These results exhibit that participation in physical exercise has a significant positive effect on both individual health status and income, supporting hypothesis 2. The test results of models (3) and (5) reveal that both δ1 and δ2 in the model are significant. The regression coefficients of physical exercise participation and individual income on SES were 0.182 and 0.105, whereas the regression coefficients of physical exercise and individual income on SES were 0.173 and 0.169, both significant at the 1% confidence interval. After including health status in the model, the coefficient of physical exercise participation decreased from 0.193 to 0.182, remaining significant but weakened, indicating that health status partially mediates the relationship between physical exercise and SES. After adding personal income to the model, the regression coefficient of physical exercise decreased from 0.193 to 0.173, indicating that income also serves as a partial mediating pathway (δ1 < *α*). This finding supports Hypothesis 3, indicating that participation in physical exercise can improve an individual’s SES by improving health and income. The total effect of physical exercise on SES was 0.193, with the mediating effect of health calculated as 0.016 (0.156 × 0.105). This means that 8.4% (0.01638/0.193) of the effect of physical exercise on SES is achieved through improved health. The mediating effect of individual income was 0.038 (0.223 × 0.169), indicating that 19.5% (0.03768/0.193) of the effect of physical exercise on SES is achieved through increased income. Health status and individual income play key mediating roles in how physical exercise affects SES, functioning synergistically rather than independently. Improved health provides a physical foundation for individuals to access more job opportunities and improve work efficiency, leading to higher income. In turn, increased income creates better conditions for individuals to engage in exercise, further improving their health. Overall, these factors have a combined effect on enhancing individual SES.

### 4.5 Model diagnostics and robustness test

To prevent high correlations among variables from undermining the estimation accuracy of the regression model, this study adopts equation-level multicollinearity diagnostics. Specifically, for each empirically estimated equation (baseline, +health, +income), VIF/GVIF values were calculated separately, and for multi-level categorical variables, GVIF^(1/(2·Df)) was used as the comparative metric. The judgment thresholds follow commonly used standards in the literature: a strict threshold of <5 and a lenient threshold of <10 are considered acceptable. All categorical variables were treated as factors, while continuous variables (such as age, number of properties, health status, and the logarithm of individual income) were incorporated into the model as numerical values. The handling of missing values for VIF was consistent with that of the corresponding equation.

The results (Table 8) indicate that the GVIF^(1/(2·Df)) values for all three models remained at very low levels: approximately 1.01–1.29 for the baseline model (with the maximum value for age ≈1.29); approximately 1.01–1.34 after adding health status (with relatively higher values for age ≈1.34 and health status ≈1.12); and approximately 1.01–1.35 after adding the logarithm of individual income (with relatively higher values for age ≈1.35 and the logarithm of individual income ≈1.33). Other variables also had values well below the thresholds, including household registration (≈1.18–1.23), political affiliation (≈1.10–1.11), and participation in physical exercise (≈1.10). Multi-level factors (education level Df = 4 ≈ 1.11, household economic status Df = 4 ≈ 1.04–1.05, social class Df = 9 ≈ 1.02, region Df = 2 ≈ 1.03–1.05) were similarly within safe ranges.

**Table 8:** Multicollinearity test results of the frequency of physical exercise to improve SES.

Variable	Df	Benchmark Model		+Health status		+Personal income	
		GVIF	GVIF^ (1/(2*Df))	GVIF	GVIF^ (1/(2*Df))	GVIF	GVIF^ (1/(2*Df))
Physical exercise	1	1.21	1.10	1.21	1.10	1.21	1.10
Genders	1	1.04	1.02	1.05	1.02	1.07	1.03
Household registration	1	1.40	1.18	1.40	1.18	1.50	1.22
Age	1	1.67	1.29	1.79	1.34	1.82	1.35
Marriage	1	1.06	1.03	1.06	1.03	1.07	1.03
Member of the Chinese Communist Party	1	1.22	1.10	1.22	1.10	1.22	1.11
Educational attainment	4	2.31	1.11	2.32	1.11	2.38	1.11
Household economic status	4	1.40	1.04	1.44	1.05	1.43	1.05
Number of properties	1	1.05	1.03	1.05	1.03	1.05	1.03
Social hierarchy	9	1.39	1.02	1.41	1.02	1.40	1.02
Basic medical insurance	1	1.02	1.01	1.02	1.01	1.02	1.01
Region	2	1.15	1.03	1.15	1.04	1.21	1.05
Health status	1			1.24	1.12		
Personal income	1					1.77	1.33

These results indicate that the linear correlations among the explanatory variables are extremely weak, with no concerning multicollinearity present. Consequently, the standard errors of the parameter estimates are not significantly inflated by collinearity, confirming the reliability of the regression results.

Reliable empirical results form the basis for the conclusions in this study. To ensure reliability, this study adopted Zhong, H’s robustness test method (48), replacing the binary participation variable in the benchmark regression with “frequency of physical exercise.” This approach further tests the robustness of the impact of physical exercise participation on SES. The frequency of participation is an ordinal variable, reflecting an individual’s frequency of physical exercise. The answers to A30_9 in the CGSS questions were reassigned, and the answers “never,” “several times a year or less,” “several times a month,” “several times a week,” and “every day” corresponded to 1–5 points, respectively. The results of the ordinal Ologit regression, using sets of stepwise control variables, are presented in Table 9.

**Table 9:** Robustness test results of the frequency of physical exercise to improve SES.

Variable	SES			
	(1)	(2)	(3)	(4)
Frequency of participation in physical exercise	0.178^***^	0.050^***^	0.046^**^	0.043^**^
	(11.225)	(2.792)	(2.544)	(2.337)
Health status			0.104^***^	0.087^***^
			(3.543)	(2.955)
Personal income				0.164^***^
				(6.048)
Control variables	Not control	Control	Control	Control
Prob>Chi^2^	127.355	3263.876	3276.447	3314.288
Pseudo R^2^	0.009	0.240	0.241	0.244
Observation	5,410	5,410	5,410	5,410

z-statistics in parentheses.

^*^p < 0.1, ^**^p < 0.05, ^***^p < 0.01.

In the basic model (model 1), which did not control for any variables, the impact of physical exercise frequency on SES was significantly positive (coefficient = 0.178, *p* < 0.01), indicating that higher levels of physical exercise participation were associated with a higher tendency of individuals assessing their SES more favorably. These results provide preliminary support for the robustness test. After introducing all benchmark control variables in the model (2), the coefficient for physical exercise frequency decreased slightly to 0.050, still significant within the 1% confidence level. This indicates that the social status improvement effect of physical exercise frequency remained valid after controlling for a range of socio-demographic variables and was consistent with the main model. Model (3) further controlled for individual health status, with the coefficient for physical exercise frequency decreasing slightly to 0.046, still significant within the 5% confidence interval. Health status had a significant positive impact on SES (0.104, *p* < 0.01). In model (5), after adding individual income, the coefficient for physical exercise frequency decreased further to 0.043 but remained significant within the 5% confidence interval, with income level also having a significant positive effect on SES (0.164, *p* < 0.01). The robustness test results revealed that higher frequencies of physical exercise were associated with a higher likelihood of improving individual SES, confirming the reliability of the empirical results.

## 5 Conclusion

### 5.1 Main conclusion

It is important to note that due to the cross-sectional nature of the CGSS data, the findings of this study demonstrate a significant association between physical exercise and SES, rather than definitively establishing a causal relationship. While we have controlled for a series of socioeconomic and demographic factors, the possibility of reverse causality or the presence of unobserved confounding variables cannot be entirely ruled out.

Based on data from the 2021 China Comprehensive Social Survey, this study empirically examined the effect of physical exercise on improving individual SES and its underlying mechanism. The study reached the following conclusions:

(1) Higher SES is associated with greater awareness of physical exercise and more active participation in it.
(2) Participation in physical exercise significantly increases the probability of improving individuals’ SES.
(3) The effects of physical exercise on improving SES varied by gender, urban–rural status, marital status, education level, and intergenerational factors. Male individuals, urban residents, married individuals, those with low educational qualifications, and older adults were more likely to participate in physical exercise to improve their SES.
(4) Health status and personal income serve as the intermediary channels through which physical exercise can improve individual SES. Specifically, participating in physical exercise can improve SES by enhancing health and increasing income.

### 5.2 Policy implications

(1) Strengthening the promotion of physical exercise and improving national participation awareness. The government and all sectors of society should increase the promotion of physical exercise through various channels, such as social media, public service advertisements, and community outreach activities. These efforts should disseminate the positive impact of physical exercise on social and economic status, with a particular focus on groups with low SES and limited awareness of physical exercise. Targeted promotion should guide these groups to realize that physical exercise not only benefits health but also offers opportunities for personal career development and social integration. This approach aims to improve public enthusiasm and initiative for participation in physical exercise.
(2) Optimizing the allocation of sports resources to promote the balanced development of physical exercise. Investment in sports resources should be increased in rural areas and economically underdeveloped areas, and more sports facilities should be built to suit local residents’ needs, such as small fitness plazas and multi-functional sports venues. This will lower the barriers for residents to participate in physical exercise. Additionally, policies should be formulated to encourage social forces to participate in rural and grassroots sports, provide diverse sports services, and reduce the gap between urban and rural areas, as well as across regions. This approach will make it easier for residents in different regions to participate in physical exercise and create conditions that promote the improvement of social and economic status.
(3) Promoting the integration of sports and career development to help specific groups improve their SES. For males, urban residents, married individuals, those with a low educational background, older adults, and other groups with a high probability of improving their SES through physical exercise, a special plan should be formulated to integrate sports and career development. For example, employee sports activities should be promoted within enterprises by combining physical exercise with vocational training and team building. This approach will improve employees’ physical fitness and work efficiency while also creating more promotion opportunities for them. Besides, sports-related vocational skills training should be provided for individuals with low educational qualifications, such as fitness coaches or sports venue maintenance personnel, to help them improve their economic income and social status through physical exercise and related vocational skills.
(4) Improving the incentive mechanism for physical exercise to improve the sustainability of residents’ participation. An incentive mechanism for physical exercise should be established, which rewards individuals or groups who actively participate and achieve specific results. Rewards could include honorary certificates, sports equipment, fitness course coupons, and other incentives. At the community level, a physical exercise reward fund should be established to recognize and reward residents who have exercised consistently and encourage others to participate. In enterprises, employees’ participation in physical exercise should be incorporated into performance appraisal or welfare systems, encouraging them to continue participating in physical exercise and reinforcing its positive impact on improving social and economic status.

### 5.3 Limitations and prospects

Despite its contributions, this study has several limitations that should be acknowledged.

First, as noted in the conclusion section, the cross-sectional design of the CGSS data prevents us from drawing definitive causal inferences. The observed associations, while robust, may be influenced by reverse causality or unmeasured time-invariant factors.

Second, our analysis may be affected by omitted variable bias. Potential confounders such as psychological traits, social network characteristics, and cultural values were not available in the dataset. The absence of these variables might lead to an overestimation of the true effect of physical exercise on SES.

Third, although the CGSS is a nationally representative survey, our analytical sample (N = 5,410) was obtained after listwise deletion of missing values. If the missingness of data is non-random, this approach may introduce selection bias. Additionally, the exclusion of specific provinces (e.g., Xinjiang, Tibet) restricts the generalizability of our findings to the entire Chinese population.

Fourth, the measure of physical exercise participation in this study is self-reported and binary (yes/no), which is subject to recall and social desirability bias. Meanwhile, this measure lacks information on exercise intensity, duration, and type, which could be important for understanding the mechanisms.

Future research should seek to address these limitations by utilizing longitudinal or panel data to better establish causality. Employing instrumental variable (IV) approaches could further help mitigate endogeneity concerns. Moreover, collecting more granular data on exercise patterns, while incorporating metrics related to psychological traits and social networks, would provide a more comprehensive understanding of the relationship between physical activity and SES.

## Data Availability

The original contributions presented in the study are included in the article/Supplementary material, further inquiries can be directed to the corresponding author.
